# X‐Linked Intellectual Developmental Disorder‐93 Caused by BRWD3 Mutation in Females: A Case Report and Literature Review

**DOI:** 10.1002/mgg3.70251

**Published:** 2026-06-15

**Authors:** Yang Xiu, Yige Zhang, Yake Jiao, Wei Wang, Yanyan Hu

**Affiliations:** ^1^ Binzhou Medical University Yantai People's Republic of China; ^2^ Department of Neurology Children's Hospital of Soochow University Suzhou People's Republic of China; ^3^ Shandong Second Medical University Weifang People's Republic of China; ^4^ Department of Child Health Linyi People's Hospital Linyi People's Republic of China

**Keywords:** BRWD3, case report, female, XLID93

## Abstract

**Objective:**

To report the clinical manifestations and genetic diagnosis of a female patient with X‐linked intellectual developmental disorder‐93 (XLID93, OMIM#300659) caused by a BRWD3 gene mutation. Systematically summarize the clinical and genetic characteristics of reported female cases and analyze the genotype–phenotype correlation.

**Methods:**

A case of XLID93 in a 1‐year‐and‐3‐month‐old girl was reported. Perform whole‐exome sequencing, qPCR copy number verification, X‐chromosome inactivation analysis, and three‐dimensional protein structure prediction for the child in this case; systematically search relevant databases, and summarize and analyze the phenotypes and genotypes of 5 female patients with BRWD3 mutations reported in the literature to date.

**Results:**

The patient was a 1‐year‐and‐3‐month‐old female presenting with delayed gross motor development, unable to stand or walk independently; poor fine motor skills; high muscle tone in the upper limbs and low muscle tone in the lower limbs. Her head circumference was 48.2 cm (95th—97th). She had slightly wide eye spacing, a low nasal bridge, a prominent forehead, a short neck, and a low posterior hairline. There was a heterozygous deletion variation of exon (loss1, EXON 21–30) in the BRWD3 gene of the patient. First‐generation verification showed that both parents of the proband were wild‐type; this mutation is a de novo mutation, and it is currently impossible to determine whether it is located on the paternal or maternal X chromosome. Protein structure prediction shows that this deletion results in the complete loss of a bromodomain, which may significantly affect the protein function. Through X‐chromosome inactivation testing, the inactivation ratio of the maternally derived X‐chromosome of the proband was about 89%, indicating non‐random inactivation. In summary, the analysis of the clinical and genetic characteristics of five female patients with XLID93 syndrome caused by BRWD3 gene mutations shows that their core clinical phenotypes have obvious commonalities, mainly manifested as: developmental delay (60%), neurological manifestations (60%), head circumference greater than the 95th percentile (60%), and special facial features (40%), with the overall clinical manifestations being milder than those in the male population. Notably, all three female patients who underwent X chromosome inactivation (XCI) testing exhibited skewed XCI, and the severity of the clinical phenotype was closely related to the direction and degree of XCI skewing: patients with preferential inactivation of the mutant X chromosome had milder clinical manifestations (patient 1 had mild cognitive impairment in early childhood); in contrast, patients with preferential inactivation of the normal X chromosome and active expression of the mutant X chromosome had more severe phenotypes (patient 4 had macrocephaly, special facial features, and developmental delay). This further clarifies the important significance of XCI testing in the genotype–phenotype correlation analysis of female patients with MRX93 syndrome.

**Conclusion:**

Clinicians should recommend early genetic testing for children with intellectual disability, language development delay, macrocephaly, or epilepsy. For female patients, X‐chromosome inactivation testing should also be completed. Early diagnosis can improve long‐term outcomes and prevent the birth of severely affected male children in the same family.

## Graphical abstract

A case of MRX93 in a 1‐year‐and‐3‐month‐old girl was reported, in whom a heterozygous deletion from exon 21 to exon 30 in the BRWD3 gene occurred. We conducted a literature review to explore the clinical phenotypes and genetic characteristics of MRX93 in females.

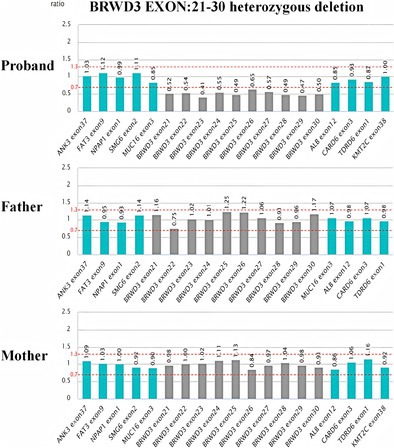

## 1 Introduction

BRWD3 (OMIM*300553) is located at Xq21.1 and encodes bromodomain and WD40 repeat‐containing protein 3 (BRWD3). This protein consists of 1802 amino acids, with a WD40 repeat domain at the N‐terminus and a bromodomain at the C‐terminus. As an epigenetic “reader” of histone acetylation, BRWD3 is involved in the regulation of chromatin remodeling, ubiquitination, and signal transduction (Field et al. 2007; Tian et al. 2022). In 2007, Field et al. first discovered and reported that variants in the BRWD3 gene can lead to X‐linked intellectual developmental disorder‐93 (XLID93, OMIM#300659). As of now, in the reported cases, the vast majority are male patients, with the following manifestations: intellectual disability (39/39), language development delay (24/25), postnatal macrocephaly (28/35), accompanied by frontal bossing (18/25) and large ears (14/26), as well as obesity (12/27) (Delanne et al. 2023). Only 4 female patients have been reported, and their phenotypes are relatively mild, mainly manifested as varying degrees of developmental delay (3/4) and epilepsy (3/4) (Delanne et al. 2023; Field et al. 2007; Tian et al. 2022). This study retrospectively analyzed the clinical phenotype and genetic testing of a female XLID 93 patient caused by a novel heterozygous deletion mutation in the BRWD3 gene, reviewed relevant literature, summarized the clinical phenotypes and genetic characteristics, and enriched the clinical phenotype spectrum of female patients with this disease.

## 2 Materials and Methods

### 2.1 Ethics Approval and Consent to Participate

This study was reviewed and approved by the Medical Ethics Committee of Linyi People's Hospital (YX200669). The parents of the proband have signed the informed consent form for the clinical study.

### 2.2 Collection of Clinical Data

One female proband aged 1 year and 3 months who visited the Department of Child Health Care of Linyi People's Hospital in December 2024 due to “discovery of gross motor delay” and her family members were selected as the research subjects. Clinical data, laboratory tests, and imaging results of the proband and her family members were collected.

### 2.3 Whole‐Exome Sequencing and Variant Analysis

Entrusted Chigene (Beijing) Translational Med Research Center Co. Ltd. to conduct trio whole‐exome sequencing. CNV analysis was performed using ML‐ExonCNV (v1.0) (Yang et al. 2026). The algorithm adopted the read depth (RD) strategy: after alignment to the human reference genome hg38 (transcript: NM_153252), the raw read count (RC) of the GRCh38.p14 virtual target region (200 bp CDS interval) was calculated by mosdepth (v0.2.6); GC bias was corrected via loess regression; normalization was performed based on Pearson's correlation (*r* ≥ 0.95) with an internal control library (> 200 WES samples); preliminary results were generated through the *Z*‐test (|*Z*| > 1.96). “Highly reliable” deletions were screened using the XGBoost expert model, followed by TaqMan probe‐based qPCR validation with ACTB as the internal reference. The consistency between qPCR results and ML‐ExonCNV detection reached 99.4%. We also conducted an X chromosome inactivation experiment using the methylation‐sensitive restriction enzyme digestion product amplification combined with capillary electrophoresis detection method. In the experiment, the criteria adopted were as follows: a ratio of inactivation of the paternal or maternal X chromosome of < 70% was defined as random inactivation, a ratio of > 70% and < 90% was considered to have moderate inactivation skewing, and a ratio of > 90% and above was considered to have extreme inactivation skewing.

### 2.4 Biological Information Analysis Methods

The AlphaFold Protein Structure Database website was used to query the three—dimensional structure of the BRWD3 protein. The location of the domain was determined based on the information from the NCBI database (http://www.ncbi.nlm.nih.gov). The Chimera X software was used to visualize the three—dimensional structures of the wild—type and mutant proteins. According to the relevant genetic variant classification criteria and guidelines of the American College of Medical Genetics and Genomics (ACMG), a comprehensive analysis of the variant sites was carried out to judge their pathogenicity.

### 2.5 Search Strategy

A systematic literature search was performed up to April 2026 across multiple electronic databases, including PubMed, Web of Science, Embase, Scopus, SpringerLink, and Cochrane Library, to identify all published cases associated with BRWD3 pathogenic variants in female individuals. No restrictions on publication date or language were applied. The search strategy combined the following keywords and synonyms using Boolean operators: “BRWD3” OR “MRX93” OR “X‐linked intellectual disability” OR “XLID” OR “mental retardation X‐linked 93” OR “BRWD3 mutation” OR “BRWD3 deletion” OR “BRWD3 variant” AND “female” OR “women” OR “girl.” All retrieved articles were manually screened by title and abstract, and full texts were reviewed to confirm eligibility. Only genetically confirmed female cases with pathogenic or likely pathogenic BRWD3 variants were included in the present study.

## 3 Results

### 3.1 Case Description

A 1‐year‐and‐3‐month‐old female presented for medical consultation due to “discovery of gross motor delay.” The child was the firstborn of the first pregnancy, delivered vaginally at a gestational age of 33^+6^ weeks, with a birth weight of 1860 g. There was no history of asphyxia at birth. The child was hospitalized in the neonatal department for 21 days, and the milk intake catch‐up was satisfactory after discharge. There was no previous history of convulsive seizures. The child successively suffered from pneumonia, conjunctivitis, rhinitis, and otitis externa between 11 and 13 months of age. Her parents denied consanguineous marriage and were in good health. The proband was able to lift the head at 4 months, turn over at 6 months, sit unsupported for a few seconds with forearm support at 7 months, and actively grasp objects. At 12 months, she could sit unsupported, crawl, flexibly change positions, and perform the pincer grasp with the thumb and index finger but could not use the pincer movement. The parachute reflex appeared at 13 months. At 7 months, she could follow visual and auditory stimuli, smile in response to teasing, and utter single vowels. At 12 months, she could say “mama” and “nannan” and understand simple nouns but could not imitate. At 14 months, she responded to her name and showed stranger anxiety. Currently at 15 months, her height is 80 cm (+0.52 SDS), weight is 10.5 kg (+0.82 SDS), and head circumference is 48.2 cm (+1.62 SDS). She cannot stand or walk independently and has poor fine motor activities, with high muscle tone in the upper limbs and low muscle tone in the lower limbs.

**FIGURE 1:**
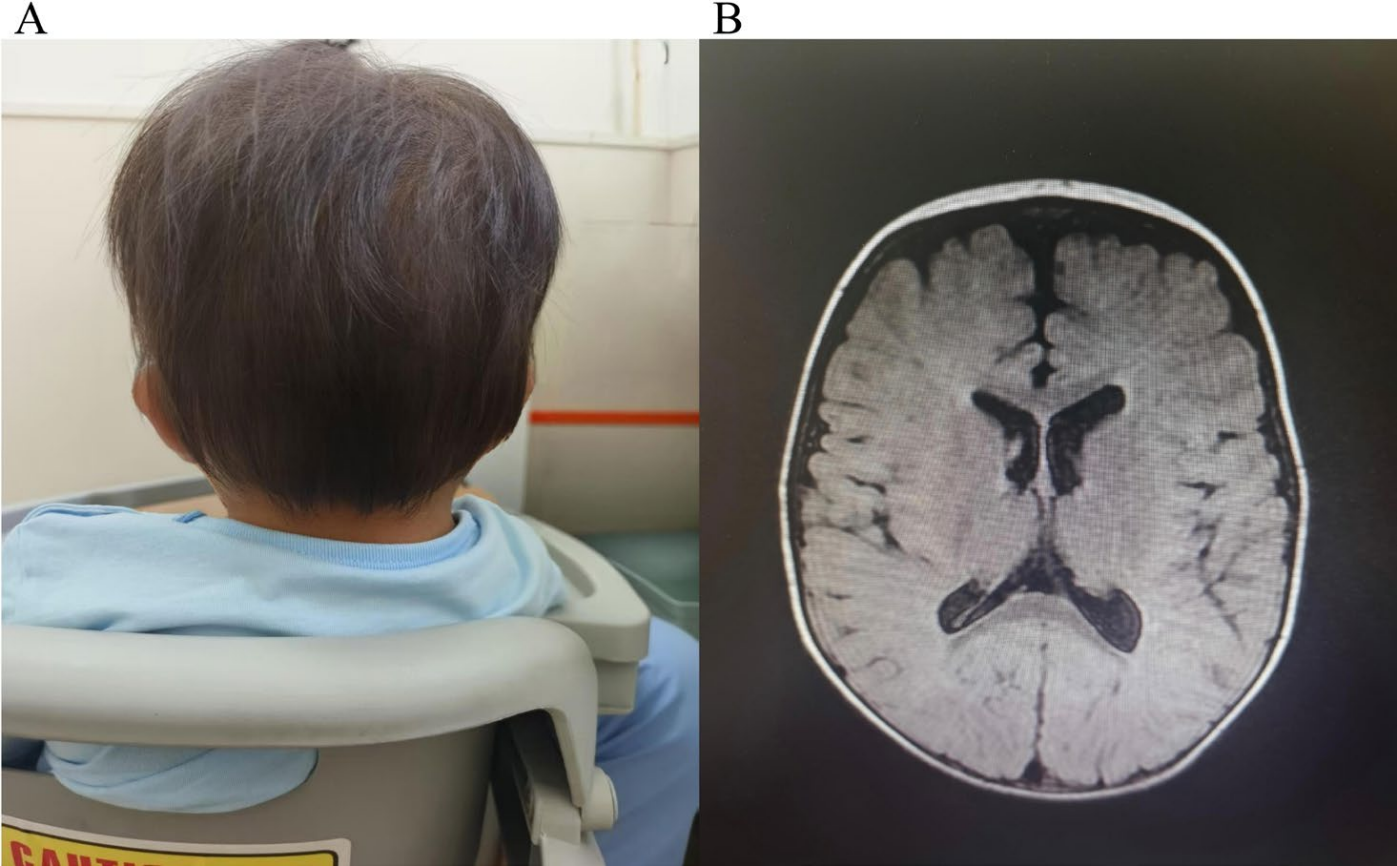
Analysis of facial features and MRI characteristics in the XLID93 proband. (A) Clinical photograph of the patient showing short neck and low posterior hairline. (B) MRI scans of the proband's brain reveal a few patchy slightly hyperintense signals on T2Flair around the anterior and posterior horns of the bilateral ventricles, and the bilateral ventricles were slightly widened.

Physical examination: The child was in good nutritional status, with slightly wide interocular distance, a low‐bridge nose, a prominent forehead, a short neck, and a low posterior hairline (Figure 1A). Physical examination of the heart, lungs, and abdomen was normal. She was in an autonomous position with normal limb movement.

Auxiliary examinations: Gesell Developmental Schedules (at 7 months of age): the gross motor behavior score was 59, the fine motor behavior score was 74, the adaptive behavior score was 65, the language behavior score was 68, and the personal‐social behavior score was 62. Gesell Developmental Schedules (at 1 year and 1 month of age): the gross motor behavior score was 80, the fine motor behavior score was 66, the adaptive behavior score was 72, the language behavior score was 71, and the personal‐social behavior score was 71. Chromosome examination: 46, XX. Brain MRI: There were a few patchy slightly hyperintense signals on T2Flair around the anterior and posterior horns of the bilateral ventricles, and the bilateral ventricles were slightly widened (Figure 1B). Echocardiogram: Patent foramen ovale. Video electroencephalogram showed no abnormalities. As shown in Table 1, the patient presented global developmental delay.

**TABLE 1:** Clinical manifestations and developmental timeline of the patient.

Time point	Clinical and auxiliary findings
2024.04.23 (Birth)	G1P1, 33 + 6 weeks, vaginal delivery, BW 1860 g, no perinatal asphyxia
3 months	Normal brain MRI; normal fundus screening; echocardiogram: PFO
4 months	Head control achieved
6 months	Rolling over achieved
7 months	Sit briefly with forearm support; active grasping; visual & auditory tracking; social smile; single vowel sounds; Gesell: GM 59, FM 74, Adaptive 65, Language 68, PS 62
9 months	Unstable sitting; no vocalization of “mama/baba”; Gesell: GM 77, FM 82, Adaptive 78, Language 67, PS 77
12 months	Independent sitting; crawling; position shifting; immature pincer grasp; Uttered “mama” “nannan”; understood simple nouns; no imitation
13 months	Parachute reflex present; Gesell: GM 80, FM 66, Adaptive 72, Language 71, PS 71
14 months	Responded to name; stranger anxiety
15 months	Height 80 cm, HC 48.2 cm (+1.62 SDS); unable to stand/walk independently; poor fine motor; upper limb hypertonia, lower limb hypotonia; Brain MRI: Periventricular patchy T_2_‐FLAIR hyperintensities, bilateral ventricles mildly dilated; Karyotype: 46, XX

Abbreviations: BW, birth weight; FM, fine motor; G1P1, Gravida 1, Para 1; GM, gross motor; HC, head circumference; MRI, magnetic resonance imaging; PFO, patent foramen ovale; PS, personal‐social.

### 3.2 Genetic Testing Results

**FIGURE 2:**
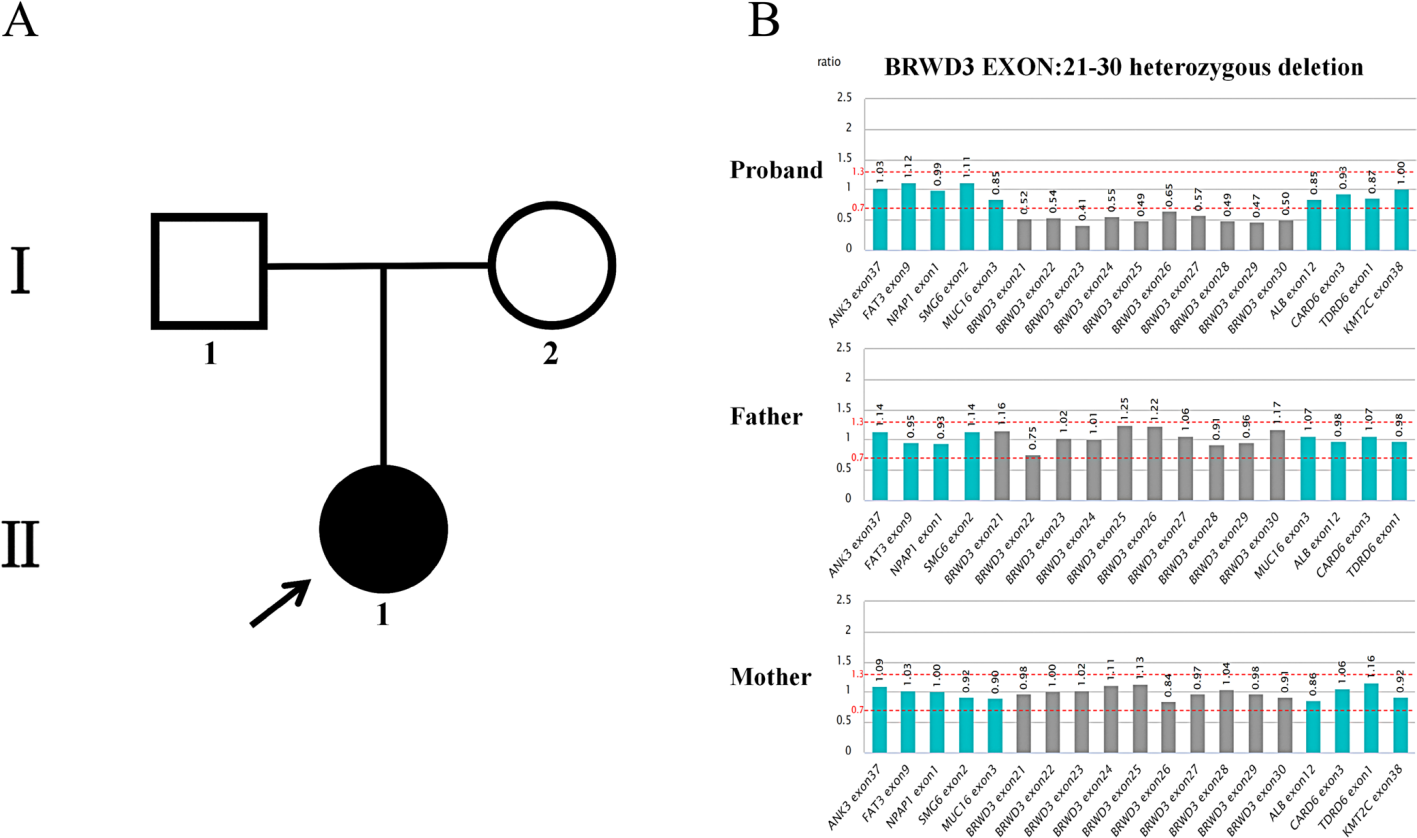
Pedigree analysis of Chinese families with a novel BRWD3 mutation. (A) The figure illustrates the pedigree of a family affected by X‐linked intellectual developmental disorder‐93 (XLID93). Unaffected males are represented by blank squares, unaffected females are represented by blank circles, and confirmed XLID93 patients by filled black circles. The proband is marked with a black arrow. (B) Copy number analysis of the BRWD3 gene in the proband and her parents based on next‐generation sequencing (NGS) data. The *y*‐axis represents the relative read depth ratio (normalized to normal controls, scale range: 0–2.5). The *x*‐axis lists the tested exonic loci, with cyan bars indicating reference control exons and gray bars indicating exons 21–30 of the BRWD3 gene. The red dashed lines mark the diagnostic thresholds: a ratio < 0.7 indicates heterozygous deletion (1 copy), a ratio > 1.3 indicates duplication (3 copies), and a ratio between 0.7 and 1.3 indicates normal diploidy (2 copies). The proband shows a heterozygous deletion of BRWD3 exons 21–30 (all gray bars with ratios < 0.7), while both parents have normal copy numbers for all tested BRWD3 exons (ratios within the normal range of 0.7–1.3).

**FIGURE 3:**
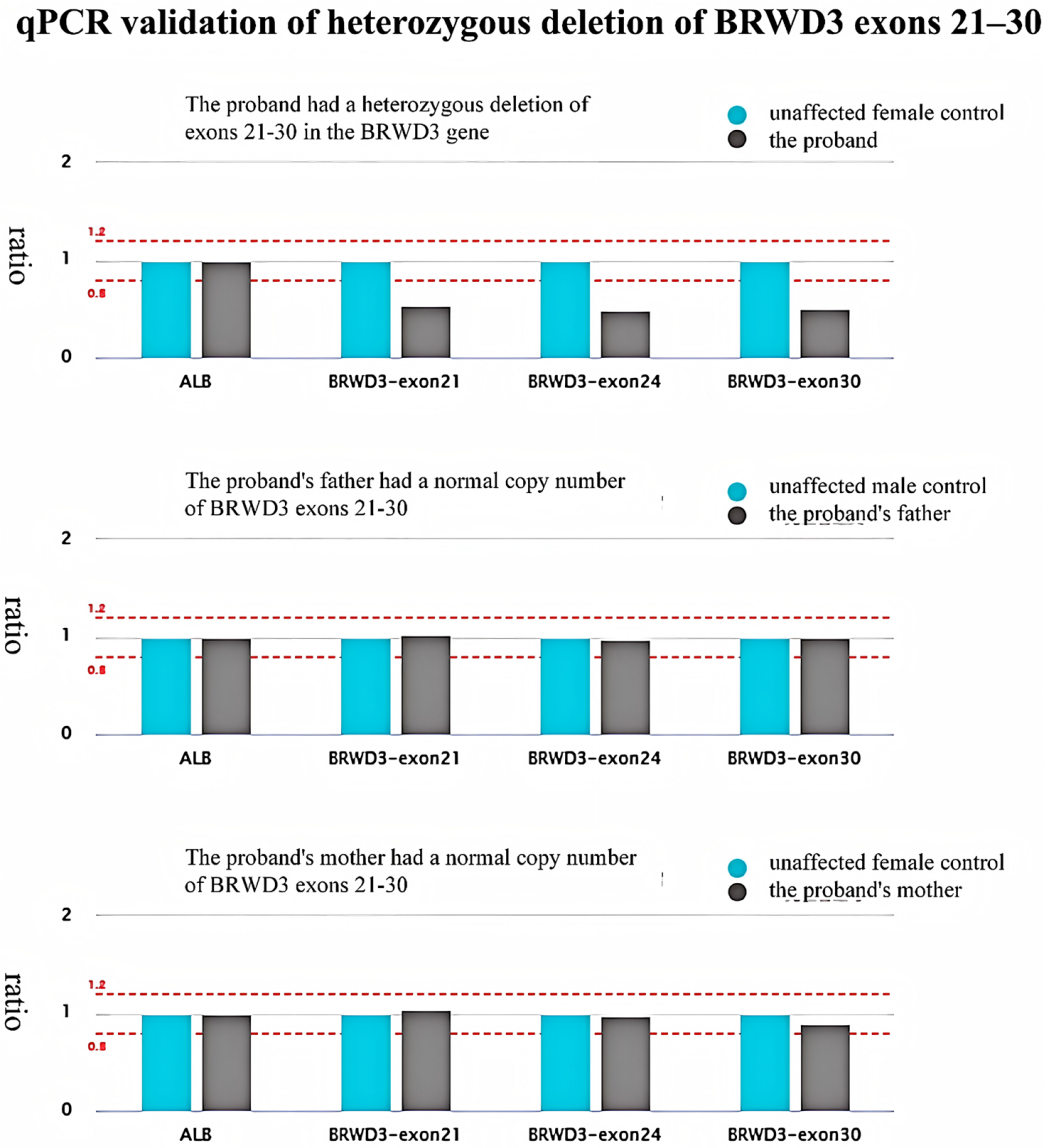
Relative copy number ratios of BRWD3 exons 21, 24, and 30 (normalized to the ALB reference gene) are shown for the proband, her father, and her mother, compared to unaffected controls. Ratios ~0.6–0.7 indicate heterozygous deletion in the proband, while ratios ~1 confirm normal copy numbers in both parents.

Through trio whole‐exome sequencing (trio‐WES), a heterozygous deletion variant was identified in the BRWD3 gene of the proband: [NM_153252.5 (BRWD3): loss (EXON: 21–30)]. Verified by NGS‐based copy number analysis, both parents of the proband exhibited no copy number abnormalities in the corresponding region, indicating that this exon deletion variant was a de novo mutation (Figure 2). This copy number variation (CNV) was further validated via orthogonal quantitative verification using TaqMan probe‐based qPCR (with ACTB as the reference gene) and MLPA‐based exon copy number variation (ML‐ExonCNV) detection. The qPCR results were consistent with the ML‐ExonCNV detection outcomes, and representative quantitative data are presented in Figure 3. After X‐chromosome inactivation testing, the inactivation ratio of the maternally derived X‐chromosome in the proband is approximately 89%, indicating non‐random inactivation.

### 3.3 Results of Biological Information Analysis

**FIGURE 4:**
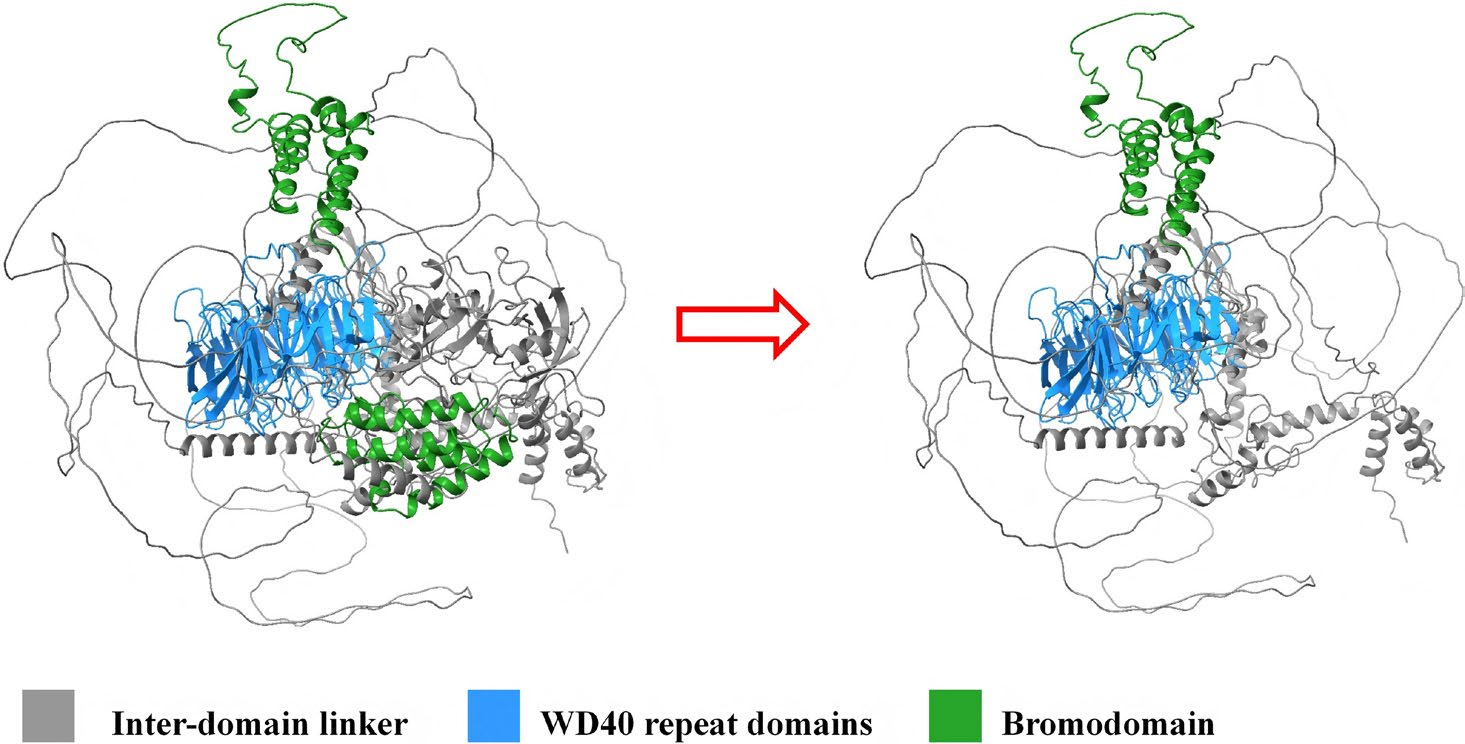
Structural impact of the large heterozygous deletion of BRWD3 exons 21–30 Schematic representation of BRWD3 protein structure before (left) and after (right) the deletion of exons 21–30. The deletion results in the complete loss of one bromodomain (green), while the WD40 repeat domains (blue) and inter‐domain linkers (gray) remain largely intact.

Based on the reference transcript NM_153252, the large fragment deletion of exons 21–30 in the *BRWD3* gene spans nucleotide positions 2732 to 3887, with an estimated deletion size of 1156 bp, corresponding to the loss of approximately 385 amino acids. This deletion results in the loss of the bromodomain in the BRWD3 protein, suggesting that this mutation is pathogenic. The structural changes between the wild‐type and mutant BRWD3 proteins are shown in Figure 4. Large fragment deletion variation of the BRWD3 gene loss (exon 21–30) may generate premature stop codons and trigger nonsense—mediated mRNA decay (NMD), leading to the loss of the bromodomain. The loss of function (LOF) of the bromodomain is the clear pathogenic mechanism (PVS1). This variation was not detected in the normal population database (PM2_Supporting). After kinship confirmation, this variation was a de novo variation in the proband (PS2_Supporting). According to the ACMG guidelines, this variation is judged as pathogenic (PVS1 + PM2_Supporting + PS2_Supporting).

### 3.4 Review of XLID 93 Patients With BRWD3 Mutations

**FIGURE 5:**
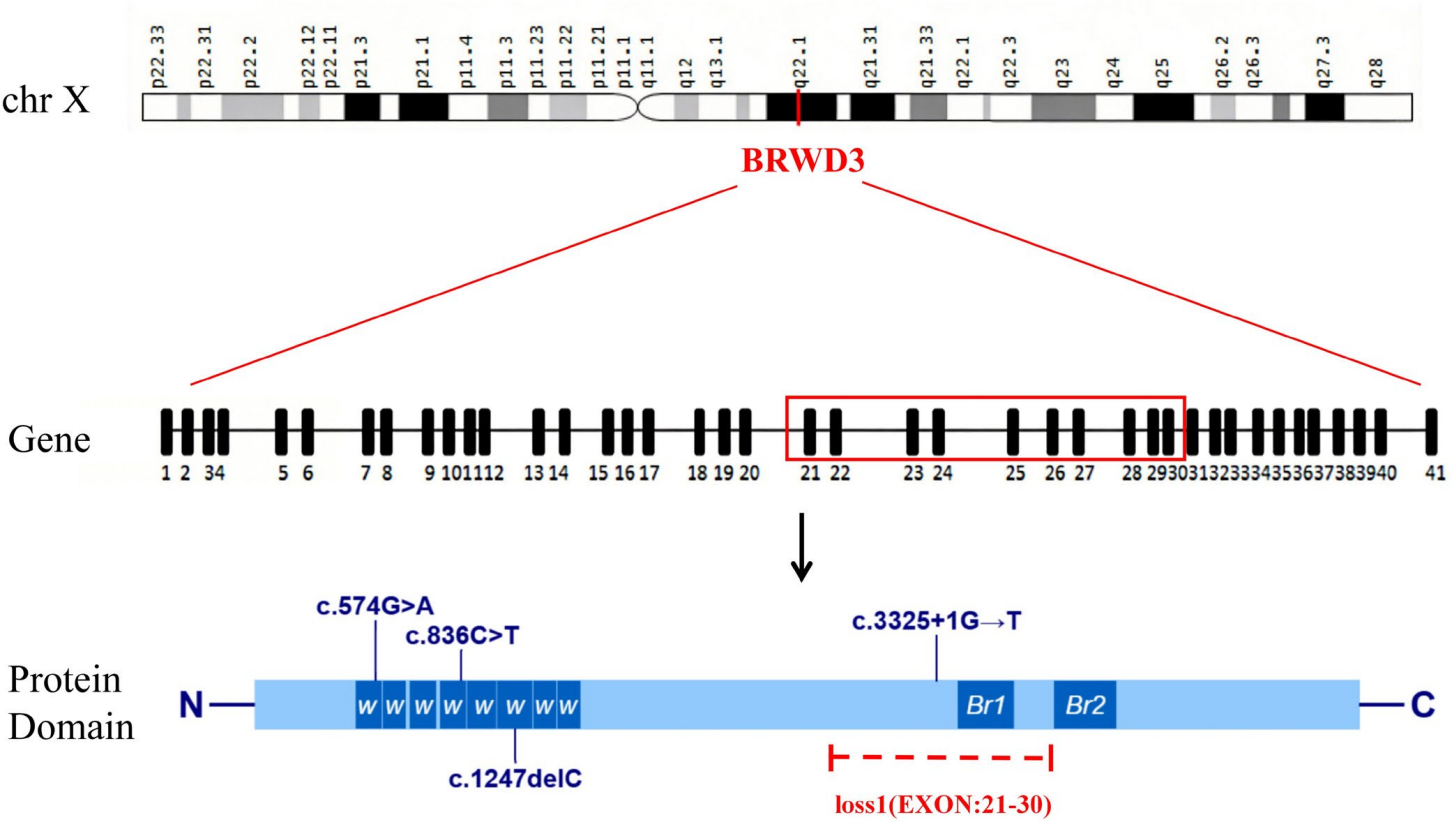
Schematic of BRWD3 mutations in female patients The diagram shows the X‐chromosomal locus, gene structure, and protein domain architecture of BRWD3, with the positions of five reported mutations. The red label indicates the large heterozygous deletion of exons 21–30 in the present study, while blue labels denote previously reported point mutations and frameshift variants in female patients. W: WD40 repeat domains; Br: Bromodomain; the dotted line represents the large‐fragment deletion in the cases of this study.

To analyze the clinical phenotypes and genotypes of female patients with BRWD3 gene mutations, we summarized the BRWD3 gene variants reported in the literature that cause XLID 93 disease. As of now, a total of 5 female cases have been reported in the literature (Table 2), including 1 splice‐site mutation + frameshift mutation, 1 frameshift mutation, 2 missense mutations, and a large‐fragment deletion in the proband of this study (Figure 5). The earliest female case was reported by Field et al. (2007). In the same family of this female patient, there were 2 female carriers and 2 male patients. This patient only had mild cognitive impairment in early childhood, and her phenotype was significantly milder than that of male patients in the same family. Among the 3 females carrying the mutant gene in the family, all had skewed X‐chromosome inactivation, with the degree of skewing ranging from high to medium, which was 96:4, 100:0, and 86:14, respectively. The chromosome carrying the BRWD3 c.3325+1G→T mutation was preferentially inactivated. Tian et al. (2022) reported the second female patient in 2022. This report suggested that in addition to causing intellectual disability, BRWD3 gene mutations were also associated with epilepsy. This patient only had two partial epileptic seizures triggered by fever at the age of 4.5 years. EEG showed sharp waves in the right central‐temporal region, and cranial MRI showed no abnormalities. After treatment with lamotrigine (2.7 mg/kg/day), the patient did not have any more epileptic seizures. Delanne et al. (2023) summarized and reported 2 female patients in 2023. Both patients had a head circumference above the 95th percentile, speech delay, and epilepsy, and they were the patients with the more severe clinical phenotypes among the reported female cases. In patient 4, the X‐chromosome inactivation was completely skewed, and all active X‐chromosomes carried the mutant allele, which might explain her more severe phenotype.

**TABLE 2:** Summary of clinical and genetic characteristics of 5 female patients with BRWD3 mutations reported in the literature.

Patient	P1 (Field et al. 2007)	P2 (Tian et al. 2022)	P3 (Delanne et al. 2023)	P4 (Delanne et al. 2023)	P5 (This example)
Age at examination	NA	NA	13 years	14 years	15 months
BRWD3 Gene Mutation	c.3325+1G→T p.W1089CfsX4	c.836C>T; p. Thr279Ile	c.1247delC；p.Ser416Leufs*11	c.574G>A；p.Gly192Arg	Loss of exons 21–30
Variant type	Splice‐site mutation + Frameshift mutation	Missense mutation	Frameshift deletion	Missense mutation	Large heterozygous deletion
Skewed X‐chromosome inactivation	94:4 (Mutant gene is preferentially inactivated)	NA	NA	100:0 (Normal gene is preferentially inactivated)	89:11
OFC at examination (cm)	NA	NA	56.7 (> 97th)	57 (95th–97th)	48.2 (95th–97th)
Facial features	—	—	—	Facial asymmetry, flat profile, bulbous nose with protruding root, thin lips, dental malposition	Prominent forehead, slightly wider eye spacing, low nasal bridge
Neurological manifestations	—	Two episodes of febrile seizures.	Primary generalized epilepsy	Seizures	—
Developmental features	Mild cognitive difficulties during early childhood	—	Started to walk at 15 months; Speech delayed; Learning disability	Started to walk at 18 months; Speech delayed; Intellectual disability	Achieved head control at 4 months of age, turned over at 6 months of age, sat up alone and started crawling at 12 months of age, parachute reflex appeared at 13 months.
Muscular hypotonia	—	—	—	+	+(lower extremity)
Other morphological features	NA	NA	—	Small and inverted toenails; cutaneous brown stains; early puberty at 8 years; ADHD、anxiety	High muscle tone in both upper extremities; short neck; low posterior hairline

Abbreviations: ADHD, Attention Deficit Hyperactivity Disorder; NA, not available; OFC, Occipital‐frontal circumference.

## 4 Discussion

BRWD3 (OMIM 300553) is located at Xq21.1 and contains 41 exons. It encodes a protein consisting of 1802 amino acids. Its N‐terminus contains 8 WD40 repeat domains, and its C‐terminus contains 2 bromodomains. Based on its structure, the putative mode of action of the BRWD3 gene can be inferred. Proteins containing WD40 repeat domains play key roles in many cellular functions, including signal transduction, protein degradation, and apoptosis (Hu et al. 2023); bromodomains are present in many chromatin‐associated proteins and most histone acetyltransferases, and they can selectively bind to acetylated lysine residues in histone tails (Gajjela and Zhou 2023).

BRWD3 was initially discovered at the t(X;11)(q13;q23) translocation breakpoint in a group of patients with B‐cell chronic lymphocytic leukemia (B‐CLL) with extremely poor prognosis (Ye et al. 2022). Functional analysis in Drosophila further confirmed that the deletion of dBRWD3 (the Drosophila homolog of BRWD3) can suppress leukemia‐like hematopoietic tumors (Fujisawa and Filippakopoulos 2017; Yu et al. 2022). In 2007, Field et al. (2007) first reported that BRWD3 gene mutations are associated with X‐linked intellectual disability with macrocephaly. In 2019, it was officially named XLID 93 syndrome, which is an X‐linked dominant genetic disease mainly characterized by intellectual disability, language development delay, macrocephaly with a prominent forehead, and other special facial features.

In Drosophila experiments, dBRWD3 plays a role in the Janus tyrosine kinase/signal transducer and activator of transcription (JAK/STAT) pathway. The JAK/STAT pathway is pleiotropic in Drosophila development and evolutionarily conserved, and its functions include not only cell proliferation and hematopoiesis but also are equally important for the maintenance and proliferation of optic lobe neuroepithelial stem cells (Chen and Desplan 2020; Fujisawa and Filippakopoulos 2017; Yu et al. 2022). Prolonged exposure of neurons to brain‐derived neurotrophic factor (BDNF) activates the JAK/STAT pathway. The BDNF‐induced JAK/STAT transcriptome encompasses all major classes of ion channels and neurotransmitter receptors in the brain, as well as key regulators of synaptic plasticity, neurogenesis, and axonal remodeling. The neuronal BDNF‐JAK/STAT pathway forms a non‐classical mechanism, and this signaling network regulates numerous genes associated with epilepsy syndromes (Hixson et al. 2019). This may explain the reasons for the varying degrees of developmental delay and the high incidence of epilepsy syndromes in patients with BRWD3 gene mutations.

The incidence of XLID93 is higher in males than in females. As of now, 56 cases have been reported, including 52 male cases and only 4 female cases. The phenotypes in females are relatively mild and are easily overlooked in clinical diagnosis and treatment. So far, there has been no summary report. In the early stage of development, to balance the expression dosage of X‐linked genes between the two sexes, female cells adopt the X chromosome inactivation (XCI) compensation mechanism. That is, one of the two X chromosomes undergoes transcriptional silencing, and the probability of inactivation of the paternal X (Xp) and the maternal X (Xm) is about 50% each so as to achieve epigenetic regulation. Many diseases are associated with XCI escape and skewing, and the symptoms and severity of these diseases largely depend on the status of XCI (Furlan and Galupa 2022; Sun et al. 2022). Not all genes on the inactive X chromosome (Xi) are in a suppressed state. It is estimated that about 15%–30% of Xi genes can escape gene suppression and be expressed (Balaton and Brown 2016; Carrel and Willard 2005; Loda et al. 2022; Oliva et al. 2020). The existence of escape genes has been found to be crucial in the pathogenesis of many diseases, including autoimmune diseases and cancer (Nascimento 2019; Shi et al. 2024; Winham et al. 2019). Skewed X‐chromosome inactivation (XCI) is defined as the preferential inactivation of one parental X chromosome in ≥ 75% of nucleated cells. Because the choice of which X will become the Xi is normally random and occurs early in gastrulation, extreme deviations can arise when selection falls within the tail of a normal distribution or when cells carrying a particular parental allele possess a survival or proliferative advantage (Sun et al. 2022). Population studies estimate that 1.5%–23% of females exhibit skewing (BIOS consortium et al. 2019; Sun et al. 2022) and both its degree and direction can modulate phenotypic severity.

The test results of the children in this study showed that the X‐chromosome inactivation ratio was 89:11. The inactivation ratio of the maternally derived X‐chromosome in the proband was 89%, indicating non‐random inactivation, which met the judgment criteria for skewed XCI. After confirmatory qPCR testing of the parents, neither of the proband's parents carried the deletion of exons 21–30, suggesting that this variant was a de novo mutation. However, since no phase analysis or allele‐specific testing was performed in this study, it was impossible to further trace the specific parental origin of the deleted allele. It could not be clearly determined whether the X‐chromosome carrying the BRWD3 deletion was of paternal or maternal origin, and a definite conclusion of “preferential inactivation of the normal allele” could not be drawn for the time being. Among the 5 female cases summarized in this study, XCI analysis was performed in 3 cases. For patient 1, the ratio was 96:4 (with the mutant gene being predominantly inactivated), and for patient 4, it was 100:0 (with the normal gene being predominantly inactivated). In terms of phenotypes, patient 1 only had mild cognitive impairment in early childhood, while patient 4 had a more severe phenotype, including macrocephaly, special facial features, and developmental delay. Based on the association between the direction of skewed XCI and the severity of the phenotype in previous female cases, we speculated that the more significant developmental delay in this patient might be related to skewed XCI, but this inference still requires further experimental verification. All indicators showed significant skewness and were closely related to clinical manifestations. Individuals with mostly silenced mutant alleles showed milder symptoms (patient 1), while those with mostly active mutant alleles showed the most severe symptoms (patient 4). The conspicuous male predominance in published XLID93 cohorts may therefore reflect under‐recognition of mildly affected females whose skewed XCI attenuates the clinical picture, a hypothesis that warrants systematic investigation.

In this research report, the case carried a large‐fragment heterozygous deletion in exons 21–30 of the BRWD3 gene, which showed significant differences in functional impact compared with the missense mutations, frameshift mutations, and splice‐site mutations reported in previous female cases. Most of the previous female cases had point mutations or small‐fragment frameshift mutations, which usually only caused local amino acid changes or protein truncation. In most cases, part of the bromodomain and WD40 domain could be retained, and some protein functions might still remain. In this case, the large‐fragment deletion directly covered the coding region of the bromodomain, resulting in the complete loss of one of the bromodomains. It was more likely to significantly disrupt chromatin recognition and transcriptional regulation functions (Boyson et al. 2021; Fujisawa and Filippakopoulos 2017; Josling et al. 2012), and the degree of damage to the protein structure and stronger function was greater than that of most point mutations. In 2019, Ostrowski et al. (2019) reported 2 male patients carrying such mutations, both of whom presented with mild‐to‐moderate intellectual disability. In addition to the reported BRWD3‐related phenotypes, this patient also showed special facial features such as slightly wide interorbital distance, a low‐bridge nose, a short neck, and a low posterior hairline, as well as a previously unreported sign of high muscle tone in the upper limbs, further expanding the phenotypic spectrum of XLID 93.

This patient is a premature infant at 33^+6^ weeks of gestation. Head MRI shows abnormal periventricular signals and ventricular enlargement. All of the above factors may independently lead to neurodevelopmental delay. Therefore, in the phenotypic interpretation, we distinguished the clinical manifestations: macrocephaly, characteristic facial features, and low muscle tone in the lower limbsare more in line with the core phenotype of XLID93 syndrome and are more likely to be directly caused by BRWD3 functional defects, while partial motor development delay is difficult to completely rule out the confounding effects of prematurity and non‐specific brain imaging changes. At the same time, this study only detected the XCI status of peripheral blood leukocytes. The XCI pattern of peripheral blood cannot fully represent the inactivation status of neural tissues such as brain tissue, which is an important limitation in the genotype–phenotype association analysis of this study. It is worth noting that among the 4 previously reported female patients, 3 had a history of “epilepsy.” Since this patient was examined at an early stage, the Gesell Developmental Schedules had shown that the developmental quotients in multiple functional areas were consistently lower than the normal level. Given that the disease may lead to intellectual disability and epileptic seizures as the patient ages, long‐term follow‐up is still needed to clarify the long‐term neurodevelopmental outcomes.

This study has certain limitations. First, this study was only interpreted based on clinical phenotypes, genetic testing, and XCI analysis. In vitro functional experiments were not conducted to verify the specific effects of this large‐fragment deletion on the function and stability of the BRWD3 protein and the downstream JAK/STAT pathway. Therefore, the pathogenic mechanism could not be directly confirmed at the molecular level. Second, the patient was a premature infant with nonspecific brain MRI changes, which may have a confounding impact on the developmental phenotype. Finally, the peripheral blood XCI results cannot fully reflect the state of the central nervous system. All the above factors may affect the accuracy of the genotype–phenotype association.

In summary, X‐linked intellectual developmental disorder‐93(XLID93) is caused by mutations in the BRWD3 gene. Currently, there are few reported female cases. The clinical manifestations are complex and diverse, with varying degrees of severity, involving multiple systems, making clinical diagnosis quite difficult. Early diagnosis, genetic counseling, and multidisciplinary follow‐up are crucial for the treatment and prognosis of this disease. This study discovered a female patient with a large‐fragment deletion that has not been previously reported, enriching the pathogenic variants of XLID 93 and providing more clues for revealing its pathogenic mechanism. Clinically, for children with intellectual disability, language development delay, macrocephaly, or epilepsy, it is recommended to conduct genetic testing as early as possible and further analyze genes and clinical phenotypes to make a clear diagnosis.

For female patients, it is recommended to complete X‐chromosome inactivation (XCI) testing. However, when interpreting the XCI results, caution should be exercised to avoid over‐inferring the specific direction of inactivation and its causal relationship with the phenotype. Moreover, vigilance should not be relaxed due to mild phenotypes, and one should not relax vigilance due to mild phenotypes. Missed diagnosis in female patients not only delays their own intervention opportunities but may also lead to the birth of severely affected male children in the same family, significantly reducing the quality of life of the entire family. In short, regard mild‐symptom female patients as a hidden danger to family health; only through early identification and active management can the transmission chain of the pathogenic gene to severely affected males be cut off.

## Author Contributions

Y.X., Y.Z., Y.J., W.W. and Y.H. confirm the authenticity of all the raw data. Y.H. was responsible for study execution, patients screening and enrollment. Y.X. and Y.Z. participated in the patients screening and enrollment and contributed to writing of the manuscript. Y.J. and W.W. contributed to data collection of the patient. Y.J. contributed to revise the manuscript. All authors read and approved the final version of the manuscript.

## Funding

The study was supported by The School Health Association of Shandong (JKZX2024162) and National Health Commission Capacity Building and Continuing Education Center (GWJJZX20251001027).

## Ethics Statement

This study was reviewed and approved by the Medical Ethics Committee of Linyi People's Hospital (YX200669). Written informed consent was obtained from the legal guardians of the patient, who were informed that the child's clinical information and images would be published in an open access journal, that the child might be recognizable, and that full anonymization could not be guaranteed. The guardians provided signed informed consent for publication.

## Conflicts of Interest

The authors declare no conflicts of interest.

## Data Availability

The data that support the findings of this study are available on request from the corresponding author. The data are not publicly available due to privacy or ethical restrictions.
